# Latent Class Analysis of Pediatric Bronchial Foreign Body Complications and Their Associated Factors

**DOI:** 10.1002/pdi3.70074

**Published:** 2026-09-23

**Authors:** Yong Qian, Ling Wang, Chao Niu, Donghai Wang, Yetao Luo

**Affiliations:** ^1^ Department of Respiratory Medicine Children's Hospital of Chongqing Medical University National Clinical Research Center for Children and Adolescents' Health and Diseases, Ministry of Education Key Laboratory of Child Development and Disorders Chongqing China; ^2^ International Science and Technology Cooperation Base of Child Development and Critical Disorders Chongqing Key Laboratory of Pediatric Metabolism and Inflammatory Diseases Chongqing China; ^3^ Department of Infection Control Second Affiliated Hospital of the Army Medical University (Xinqiao Hospital) Chongqing China

**Keywords:** bronchial foreign body, children, complications, diagnosis‐related group, latent class analysis

## Abstract

This study aimed to provide a theoretical basis for improving early diagnosis and complication prevention by exploring the latent classes of pediatric bronchial foreign body complications, analyzing their associated factors, and assessing the impact of different latent classes on healthcare resource utilization. Children who underwent bronchoscopic foreign body removal at the Children's Hospital of Chongqing Medical University from January 1, 2018 to July 31, 2023, were retrospectively reviewed. Latent class analysis was performed on complications with an incidence > 10% (including pneumonia, pulmonary emphysema, granulation tissue, bronchial stenosis, atelectasis, and pulmonary consolidation). Logistic regression was used to identify risk factors among different latent complication classes, and comparative analyses compared healthcare resource utilization. Among the 238 included children, complications with an incidence exceeding 10% were analyzed and clustered into four latent classes: the “Pneumonia Group” (C1, 40 cases, 16.8%), the “Pneumonia‐Atelectasis Group” (C2, 33 cases, 13.9%), the “Emphysema‐Granulation tissue Group” (C3, 106 cases, 44.5%), and the “Bronchial Stenosis‐Granulation tissue Group” (C4, 59 cases, 24.8%). Results identified a history of foreign body aspiration, asymmetric breath sounds, C‐reactive protein ≥ 8 mg/L, and neutrophil percentage as associated factors (*p* < 0.05). Healthcare resource utilization was significantly lower in groups C3 and C4 regarding hospital stay, treatment costs, and antibiotic days compared to C1 and C2 (*p* < 0.05). These latent classes offer theoretical support and practical reference for early diagnosis, complication prevention and diagnosis‐related group‐based payment optimization.

## Graphical abstract

This study employed latent class analysis (LCA) to identify four potential classes of pediatric bronchial foreign body complications. Significant differences were observed among the characteristics of these classes, which were associated with factors such as a history of foreign body aspiration, use of rigid bronchoscopy, asymmetric breath sounds, C‐reactive protein (CRP) ≥ 8, and neutrophil percentage. Healthcare professionals should pay particular attention to the Pneumonia–Atelectasis Group, providing refined and individualized treatment strategies to improve clinical outcomes and reduce medical costs.

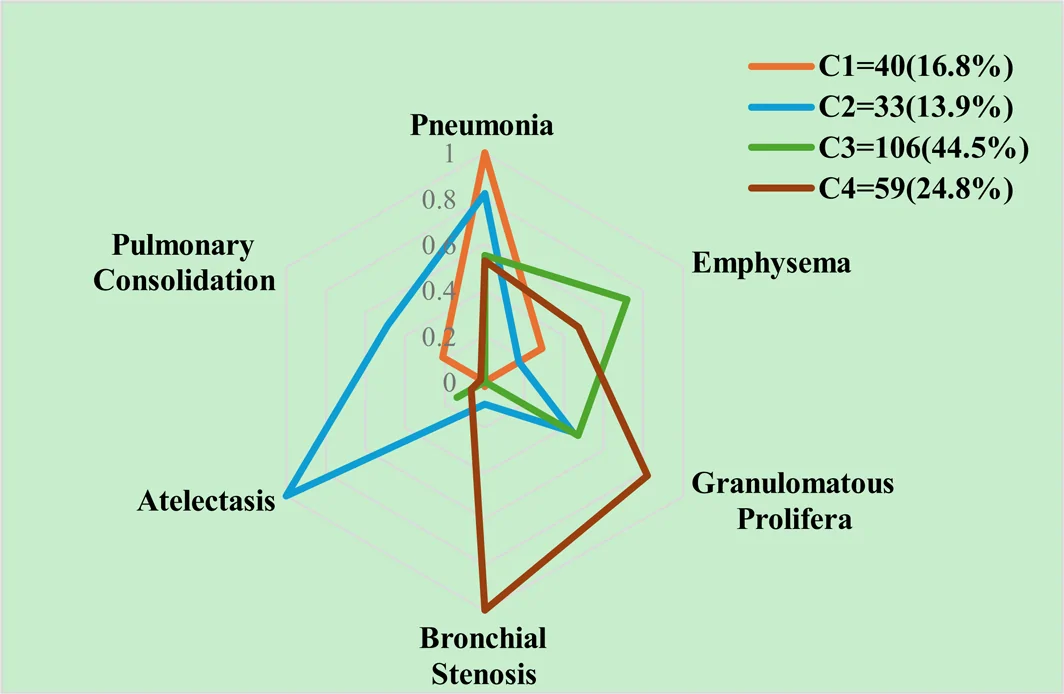

## 1 Introduction

Bronchial foreign bodies (BFBs) refer to conditions in which exogenous objects are accidently aspirated through the mouth or nose into the trachea or bronchi, potentially causing respiratory distress or even fatal asphyxiation in a short period [1, 2]. In China, the perioperative mortality rate of pediatric BFBs is approximately 2.3%, highlighting its status as a common and critical pediatric emergency [3]. BFBs exhibit distinct age‐related characteristics, most frequently occurring in children under 3 years old. They may lead to secondary complications such as pulmonary infections, pulmonary emphysema, chronic cough, recurrent wheezing, and tracheal stenosis, with severe cases being life‐threatening, imposing substantial burdens on the child's quality of life and the family's economic situation [4, 5]. Accurate identification of high‐risk children with BFBs is crucial for prevention and timely intervention. However, the occurrence of BFBs is influenced by multiple factors, including age, feeding practices, household environment, and caregiver knowledge, resulting in significant heterogeneity in risk profiles [6, 7]. Traditional research methods often rely on univariate analyses or simple scoring systems, which fail to fully capture the complexity and diversity of risk in pediatric BFBs. Latent class analysis (LCA), a statistical approach based on multivariable combinations, can effectively identify latent risk subgroups with similar characteristics, precisely locate high‐risk subgroups, and guide individualized preventive interventions, providing a novel perspective for classifying heterogeneous populations [8, 9]. This study was based on clinical data of hospitalized children with BFBs. Using latent class analysis, it explored the heterogeneous characteristics and associated factors of complications and compared the impact of different types of foreign bodies on subsequent recovery and treatment, with the aim of providing a theoretical basis for early diagnosis and prevention of complication.

## 2 Materials and Methods

### 2.1 Study Design and Subjects

This study adopted a retrospective cohort design, selecting children diagnosed with BFBs in the Respiratory Department of the Children's Hospital of Chongqing Medical University from January 1, 2018 to July 31, 2023. The inclusion criteria were as follows: (1) age 0–12 years; (2) the first diagnosis of BFBs; (3) having undergone bronchoscopic foreign body removal under intravenous compound anesthesia at our hospital; and (4) complete medical records. The exclusion criteria were as follows: (1) concurrent tracheal or bronchial tumors; (2) hematologic diseases; or (3) incomplete medical records. This study was approved by the ethics committee of Children's Hospital of Chongqing Medical University (approval number: 2024‐205). Due to the retrospective nature of the study, informed consent was waived.

### 2.2 Data Collection

General patient data, including sex, age, mode of admission, disease course, and history of foreign body aspiration, were collected. Clinical data were systematically recorded, encompassing preoperative presentations such as pulmonary signs, hematological parameters (including C‐reactive protein [CRP], procalcitonin [PCT], white blood cell count [WBC], lymphocyte [LYM] percentage, and neutrophil [NEUT] percentage), and preoperative complications. Additionally, intraoperative findings, including endoscopic observations (granulation tissue and bronchial stenosis), foreign body type, and impaction site, were documented. Postoperative status, specifically the duration of antibiotic therapy, was also recorded. Finally, healthcare resource utilization was assessed by analyzing length of hospital stay, total medical expenses, and total duration of antibiotic use.

### 2.3 Statistical Methods

A LCA was conducted to identify patterns of complications. Complications with an incidence > 10% (including pneumonia, pulmonary emphysema, granulation tissue, bronchial stenosis, atelectasis, and pulmonary consolidation) were included in the LCA and coded as binary variables (0/1). A total of five latent class models were fitted, with the number of classes ranging from 1 to 5, and model parameters were estimated using the maximum likelihood method. Model fit was evaluated using the Bayesian information criterion (BIC), adjusted Bayesian information criterion (aBIC), and Akaike information criterion (AIC), with smaller values indicating better fit. The optimal number of latent classes was determined based on the following criteria: mean posterior probability for each class should be > 0.7 and entropy > 0.8. Further comparison between K‐class and (K‐1)‐class models was performed using the Bootstrap likelihood ratio test (BLRT) and the Lo‐Mendell‐Rubin adjusted likelihood ratio test (LMRT). If the *p*‐values from both BLRT and LMRT for a K‐class model were < 0.05, the K‐class model was considered to fit significantly better than the (K‐1)‐class model.

Categorical data were described as counts and percentages, and comparisons between groups were performed using the Chi‐square test or Fisher's exact test. Quantitative data with skewed distribution were presented as median and interquartile range (IQR), and comparisons between groups were conducted with the Kruskal–Wallis *H* test. Pairwise comparisons were performed using the Dunn–Bonferroni method to adjust *p*‐values. Based on clinical knowledge, variables potentially influencing the latent class distribution were screened. The variable combination was subsequently optimized through bidirectional stepwise regression, and the final set of variables was incorporated into an unordered logistic regression model for analysis. The variable combination was subsequently optimized through bidirectional stepwise regression, and the final set of variables was incorporated into an unordered logistic regression model for analysis. Data processing and latent class analysis were performed using Statistical Analysis System (SAS) 9.4 and Mplus 8.3 software, respectively.

## 3 Results

### 3.1 Clinical Characteristics of the Patients and Occurrence of Bronchial Foreign Body Complications

A total of 238 children diagnosed with BFBs were included in this study. The median (interquartile range) age of the patients was 19 (15, 24) months, with 157 males (66.0%). The age of children with BFBs was predominantly concentrated in the 13–24 months range (160 cases, 67.2%). The most common type of foreign body was nuts (166 cases, 69.7%). The vast majority of children (184 cases, 77.3%) had a confirmed history of foreign body aspiration. Clinically, 64.7% of the children presented with asymmetric breath sounds. Details are shown in Table 1.

**TABLE 1:** Clinical data and latent classes of pediatric bronchial foreign body complications.

Variable	Total (*n* = 238)	C1 (*n* = 40)	C2 (*n* = 33)	C3 (*n* = 106)	C4 (*n* = 59)	*p*
Sex, *n* (%)						0.85
Male	157 (66.0)	28 (70.0)	23 (69.7)	69 (65.1)	37 (62.7)	
Female	81 (34.0)	12 (30.0)	10 (30.3)	37 (34.9)	22 (37.3)	
Age (months), *n* (%)						< 0.01
≤ 12	27 (11.3)	9 (22.5)	2 (6.1)	12 (11.3)	4 (6.8)	
13–24	160 (67.2)	19 (47.5)	18 (54.5)	78 (73.6)	45 (76.3)	
> 24	51 (21.4)	12 (30.0)	13 (39.4)	16 (15.1)	10 (16.9)	
Course of disease (days), median (IQR)	10 (3, 30)	9 (3, 21)	15 (7, 30)	9 (4, 20)	10 (5, 30)	0.08
Method of admission, *n* (%)						< 0.01
Outpatient clinic	107 (45.0)	21 (52.5)	22 (66.7)	40 (37.7)	24 (40.7)	
Emergency	77 (32.3)	14 (35.0)	5 (15.1)	41 (38.7)	17 (28.8)	
Transfer from other hospitals	54 (22.7)	5 (12.5)	6 (18.2)	25 (23.6)	18 (30.5)	
Type of foreign body, *n* (%)						0.03
Nuts	166 (69.7)	21 (52.5)	19 (57.6)	81 (76.4)	45 (76.3)	
Nut shells	10 (4.2)	3 (7.5)	1 (3.0)	3 (2.8)	3 (5.1)	
Bones	9 (3.8)	2 (5.0)	0 (0.0)	5 (4.7)	2 (3.4)	
Plastic	16 (6.7)	2 (5.0)	6 (18.2)	6 (5.7)	2 (3.4)	
Others	37 (15.5)	12 (30.0)	7 (21.2)	11 (10.4)	7 (11.8)	
History of foreign body aspiration, *n* (%)						< 0.01
Denied	54 (22.7)	22 (55.0)	14 (42.4)	11 (10.4)	7 (11.9)	
Confirmed	184 (77.3)	18 (45.0)	19 (57.6)	95 (89.6)	52 (88.1)	
Site of foreign body impaction, *n* (%)						0.11
Left side	87 (36.6)	13 (32.5)	9 (27.3)	40 (37.8)	25 (42.4)	
Right side	145 (60.9)	23 (57.5)	24 (72.7)	65 (61.3)	33 (55.9)	
Both lungs/subglottic/tracheal carina	6 (2.5)	4 (10.0)	0 (0.0)	1 (0.9)	1 (1.7)	
Foreign body location (left side), *n* (%)						0.23
None	147 (61.8)	23 (57.5)	24 (72.7)	66 (62.3)	34 (57.6)	
Left main bronchus	32 (13.4)	6 (15.0)	4 (12.1)	11 (10.4)	11 (18.6)	
Distal left main bronchus	34 (14.3)	4 (10.0)	3 (9.1)	15 (14.2)	12 (20.3)	
Others	25 (10.5)	7 (17.5)	2 (6.1)	14 (13.2)	2 (3.4)	
Foreign body location (right side), *n* (%)						0.21
None	85 (35.7)	13 (32.5)	8 (24.2)	39 (36.8)	25 (42.4)	
Right main bronchus	70 (29.4)	13 (32.5)	9 (27.3)	35 (33.0)	13 (22.0)	
Right middle bronchus	49 (20.6)	5 (12.5)	9 (27.3)	19 (17.9)	16 (27.1)	
Others	34 (14.3)	9 (22.5)	7 (21.2)	13 (12.3)	5 (8.5)	
Breath sounds, *n* (%)						< 0.01
Symmetrical	84 (35.3)	24 (60.0)	9 (27.3)	32 (30.2)	19 (32.2)	
Asymmetrical	154 (64.7)	16 (40.0)	24 (72.7)	74 (69.8)	40 (67.8)	
Wheezing, *n* (%)						0.06
No	111 (46.6)	20 (50.0)	22 (66.7)	46 (43.4)	23 (39.0)	
Yes	127 (53.4)	20 (50.0)	11 (33.3)	60 (56.6)	36 (61.0)	
Crackles, *n* (%)						0.39
No	156 (65.5)	23 (57.5)	19 (57.6)	73 (68.9)	41 (69.5)	
Yes	82 (34.5)	17 (42.5)	14 (42.4)	33 (31.1)	18 (30.5)	
Three concave signs, *n* (%)						0.77
No	197 (82.8)	31 (77.5)	27 (81.8)	90 (84.9)	49 (83.1)	
Yes	41 (17.2)	9 (22.5)	6 (18.2)	16 (15.1)	10 (16.9)	
CRP (mg/L), *n* (%)						< 0.01
< 8	182 (76.5)	26 (65.0)	19 (57.6)	87 (82.1)	50 (84.7)	
≥ 8	56 (23.5)	14 (35.0)	14 (42.4)	19 (17.9)	9 (15.3)	
PCT (μg/L), *n* (%)						0.23
< 0.05	78 (32.8)	9 (22.5)	8 (24.2)	39 (36.8)	22 (37.3)	
≥ 0.05	160 (67.2)	31 (77.5)	25 (75.8)	67 (63.2)	37 (62.7)	
WBC (× 10^9^/L), median (IQR)	11.8 (8.8, 15.6)	11.4 (8.3, 15.7)	13.5 (10.0, 17.9)	12.0 (8.8, 15.5)	12.0 (8.7, 13.9)	0.23
LYM percentage, median (IQR)	0.5 (0.3, 0.6)	0.4 (0.3, 0.6)	0.4 (0.2, 0.5)	0.5 (0.4, 0.6)	0.6 (0.4, 0.7)	< 0.01
NEUT percentage, median (IQR)	0.5 (0.4, 0.7)	0.5 (0.4, 0.7)	0.6 (0.5, 0.8)	0.4 (0.3, 0.6)	0.5 (0.3, 0.7)	< 0.001

*Note:* C1, Pneumonia Group; C2, Pneumonia–Atelectasis Group; C3, Emphysema–Granulation tissue Group; C4, Bronchial Stenosis–Granulation tissue Group.

Abbreviations: CRP, C‐reactive protein; IQR, interquartile range; LYM, lymphocyte; NEUT, neutrophil; PCT, procalcitonin; WBC, white blood cell count.

All children developed at least one complication. The overall spectrum and incidence rates of complications are presented in Table 2. Complications with an incidence greater than 10% were as follows: pneumonia in 158 cases (66.4%), pulmonary emphysema in 121 cases (50.8%), granulation tissue in 111 cases (46.6%), bronchial stenosis in 61 cases (25.6%), atelectasis in 51 cases (21.4%), and pulmonary consolidation in 26 cases (10.9%).

**TABLE 2:** Occurrence of pediatric bronchial foreign body complications (*n* = 238).

Complications	Rank	Number of cases	Rate (%)
Pneumonia	1	158	66.4
Pulmonary emphysema	2	121	50.8
Granulation tissue	3	111	46.6
Bronchial stenosis	4	61	25.6
Atelectasis	5	51	21.4
Pulmonary consolidation	6	26	10.9
Mediastinal emphysema	7	8	3.4
Subcutaneous emphysema	8	4	1.7
Mediastinal shift	9	3	1.3
Pneumothorax	9	3	1.3

### 3.2 Results of Latent Class Analysis for Pediatric Bronchial Foreign Bodies

LCA was performed on BFB complications with an incidence greater than 10%. A total of five different latent class models were fitted. The LMRT for the 5‐class model was not statistically significant, indicating that the 4‐class model was preferable. The entropy index for the 3‐class LCA model was < 0.8. Therefore, the 4‐class LCA model was selected for this study. Among the four classes, Group C1 included 40 cases (16.8%), Group C2 included 33 cases (13.9%), Group C3 included 106 cases (44.5%), and Group C4 included 59 cases (24.8%). The mean posterior probabilities for assigning cases to their respective latent classes were 0.8, 0.9, 0.9, and 1.0, and the entropy index was 0.83, indicating reliable classification results for the 4‐class model. Details are provided in Supporting Information S1: Table S1.

**FIGURE 1:**
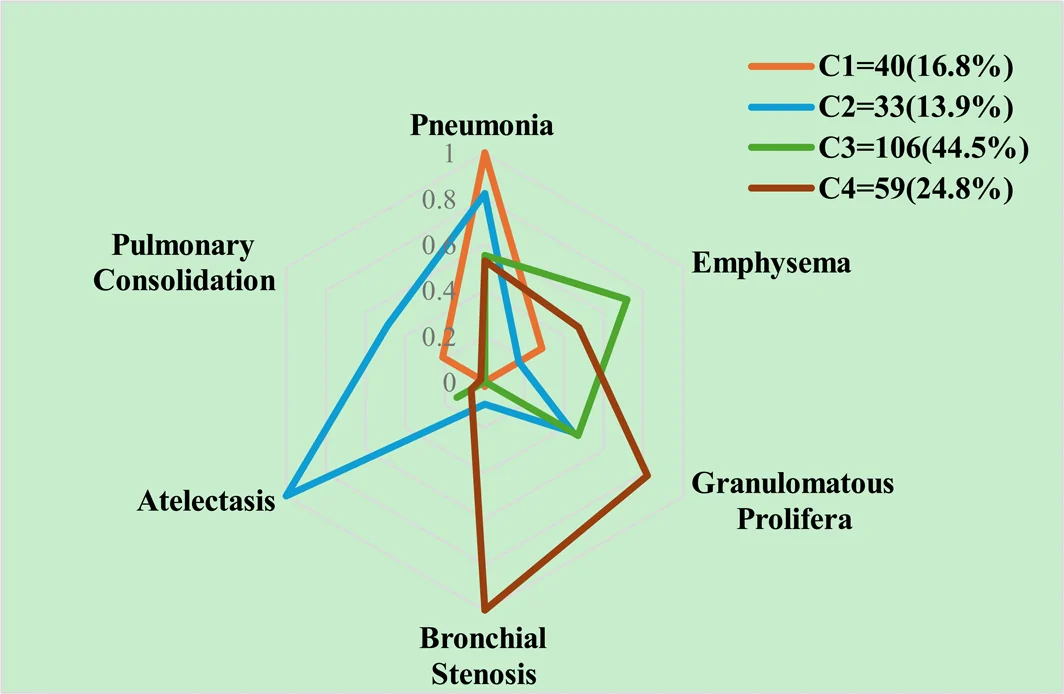
Distribution of the four latent classes for the characteristics of pediatric bronchial foreign body complications (the scale 0–1 representing the probability of complications).

Based on the LCA results and the characteristics of each group, the classes were named as follows: Group C1, where pneumonia was the predominant complication, was designated as the “Pneumonia Group”; Group C2, characterized by a high incidence of both pneumonia and atelectasis, was designated as the “Pneumonia‐Atelectasis Group”; Group C3, characterized by a high incidence of pulmonary emphysema and granulation tissue, was designated as the “Emphysema‐Granulation tissue Group”; and Group C4, characterized by a high incidence of bronchial stenosis and granulation tissue, was designated as the “Bronchial Stenosis‐Granulation tissue Group.” The distribution of the four latent classes for pediatric BFB complication characteristics is illustrated in Figure 1.

### 3.3 Clinical Data and Latent Classes of Pediatric Bronchial Foreign Body Complications

The results showed that the differences among the four latent classes of pediatric BFB complications were statistically significant in terms of age, method of admission, type of foreign body, history of foreign body aspiration, breath sounds, CRP, LYM percentage, and NEUT percentage. Details are shown in Table 1.

### 3.4 Unordered Multinomial Logistic Regression of the Latent Classes of Pediatric Bronchial Foreign Body Complication Characteristics

The screened variables were subsequently incorporated into an unordered multinomial logistic regression model. The coding of independent variables is shown in Supporting Information S1: Table S2. The results indicated that children with CRP ≥ 8 mg/L were more likely to be classified into the C1 and C2 groups than into the C3 or C4 groups; children older than 24 months tended to belong to Group C2; children with a history of foreign body aspiration and those aged 13–24 months were more likely to belong to Group C3 and Group C4; and children with asymmetric breath sounds were more likely to be classified into groups characterized by typical airway obstruction patterns, particularly Group C3. Compared to Group C3, Group C4 had a higher NEUT percentage. Details are presented in Table 3.

**TABLE 3:** Results of the unordered multinomial logistic regression analysis for the latent classes of pediatric bronchial foreign body complication characteristics.

Variable	C1 versus C3		C2 versus C3		C1 versus C4		C2 versus C4		C4 versus C3		C2 versus C1	
	OR (95% CI)	*p*	OR (95% CI)	*p*	OR (95% CI)	*p*	OR (95% CI)	*p*	OR (95% CI)	*p*	OR (95% CI)	*p*
Age (reference ≤ 12 months)												
13–24 months	0.3 (0.1, 0.9)	0.03	1.6 (0.3, 8.4)	0.59	0.2 (0.0, 0.7)	0.01	0.9 (0.1, 5.7)	0.90	1.8 (0.5, 6.2)	0.35	5.4 (0.9, 30.1)	0.05
> 24 months	0.8 (0.2, 3.0)	0.73	6.4 (1.1, 38.6)	0.04	0.4 (0.1, 1.9)	0.23	3.0 (0.4, 22.3)	0.28	2.1 (0.5, 8.9)	0.30	8.1 (1.3, 50.1)	0.02
History of foreign body aspiration	0.1 (0.0, 0.3)	< 0.001	0.2 (0.1, 0.4)	< 0.001	0.1 (0.0, 0.4)	< 0.01	0.2 (0.1, 0.7)	0.01	0.7 (0.3, 2.1)	0.56	1.5 (0.5, 4.2)	0.43
Asymmetric breath sounds	0.3 (0.1, 0.7)	0.01	1.3 (0.5, 3.3)	0.61	0.4 (0.2, 0.99)	0.047	1.6 (0.6, 4.4)	0.37	0.8 (0.4, 1.6)	0.56	4.1 (1.5, 11.6)	0.01
CRP ≥ 8 mg/L	3.1 (1.2, 7.9)	0.02	4.1 (1.6, 10.6)	< 0.01	4.2 (1.4, 12.7)	0.01	5.6 (1.9, 16.9)	< 0.01	0.7 (0.3, 1.9)	0.52	1.3 (0.5, 3.8)	0.57
NEUT percentage	1.0 (1.0, 1.1)	0.94	1.0 (1.0, 1.1)	0.17	1.0 (0.9, 1.0)	0.15	1.0 (1.0, 1.0)	0.35	1.0 (1.0, 1.1)	< 0.01	1.0 (1.0, 1.1)	0.42

Abbreviation: NEUT, neutrophil.

### 3.5 Relationship Between Latent Classes of Pediatric Bronchial Foreign Body Complications and Healthcare Resource Utilization

Length of hospital stay, treatment costs, and days of antibiotic use were used as indicators of healthcare resource utilization. Children in Group C2 had the greatest healthcare resource utilization, with the longest hospital stay, the highest treatment costs, and the longest duration of antibiotic use (all *p* < 0.05). Compared with Groups C1 and C2, Group C3 had a significantly shorter hospital stay, lower treatment costs, and fewer days of antibiotic use (all *p* < 0.05). Group C4 also had significantly lower treatment costs and fewer days of antibiotic use than Groups C1 and C2, and a shorter length of hospital stay compared with Group C2 (all *p* < 0.05). Details are shown in Table 4.

**TABLE 4:** Relationship between latent classes of pediatric bronchial foreign body complications and healthcare resource utilization.

Outcome indicators	Total	C1 (*n* = 40)	C2 (*n* = 33)	C3 (*n* = 106)	C4 (*n* = 59)	*p*
Length of hospital stay (days), median (IQR)	4 (4, 6)	4 (4, 6.5)	6 (6, 8)	4 (4, 5)^a^ ^,^ ^b^	4 (4, 6)^b^	< 0.01
Treatment costs (CNY), median (IQR)	75,000 (59,000, 101,000)	91,000 (73,000, 113,000)	94, 000 (77,000, 123,000)	67,000 (55,000, 82,000)^a^ ^,^ ^b^	68,000 (58,000, 95,000)^a^ ^,^ ^b^	< 0.01
Days of antibiotic use (days), median (IQR)	2 (2, 5)	4 (4, 7)	6 (6, 8)	2 (2, 4)^a^ ^,^ ^b^	2 (2, 4)^a^ ^,^ ^b^	< 0.01

Abbreviation: IQR, interquartile range.

*Note: p* values for pairwise comparisons were adjusted using the Dunn–Bonferroni method. Quantitative data with skewed distribution were described using the median and IQR. The Kruskal–Wallis *H* test was applied for between‐group comparisons, and the Dunn–Bonferroni method was used for pairwise comparisons to adjust the *p*‐values.

^a^ Compared with C1, *p* < 0.05.

^b^ Compared with C2, *p* < 0.05.

## 4 Discussion

### 4.1 Pediatric Bronchial Foreign Body Complications Can Be Classified Into Four Latent Classes

Using latent class analysis, this study identified four distinct classes of complications in children with BFBs: the “Pneumonia Group” (C1), “Pneumonia‐Atelectasis Group” (C2), “Emphysema‐Granulation tissue Group” (C3), and “Bronchial Stenosis‐Granulation tissue Group” (C4). Pediatric BFB complications encompass a spectrum of pathophysiological responses resulting from accidental airway retention, including pneumonia, atelectasis, emphysema, and bronchial stenosis, all of which can severely compromise respiratory function and diminish quality of life in affected children [10, 11].

These four latent classes (C1–C4) exhibit distinct profiles that necessitate correspondingly differentiated treatment strategies. Management of pneumonia primarily involves anti‐infective therapy [12]; atelectasis requires prompt relief of obstruction, with fiberoptic bronchoscopy lavage and suction serving as key interventions [13]; tracheal or bronchial stenosis calls for mechanical clearance, with the choice of procedure tailored to the length and nature of the stenosis [14]; emphysema management focuses on improving pulmonary ventilation, with lung volume reduction surgery or lung transplantation considered in advanced cases [15]; and tracheal granulation tissue necessitates targeted intervention addressing the underlying cause, with refractory lesions potentially requiring surgical excision or laser ablation [16]. These findings underscore the need for individualized treatment based on complication subtype. Accordingly, identifying latent complication classes carries significant clinical implications, enabling healthcare professionals to better understand the heterogeneity of complications, formulate personalized treatment plans, optimize healthcare resource allocation, and improve patient outcomes—thereby providing a scientific foundation for clinical classification and targeted intervention.

### 4.2 Analysis of Associated Factors for the Latent Classes of Pediatric Bronchial Foreign Body Complications

Our logistic regression analysis indicates that latent class assignment reflects distinct “clinical presentation‐inflammatory response–disease progression” phenotypes: CRP ≥ 8 mg/L was significantly associated with classification into pneumonia‐dominant categories (C1/C2) and less with categories characterized by airway obstruction/granulation (C3/C4), consistent with evidence supporting the clinical discriminatory value of CRP as a marker of inflammatory/infectious burden in pediatric pneumonia studies [17, 18, 19, 20].

Furthermore, age showed clear category‐specific patterns: compared with children ≤ 12 months, those > 24 months were more likely to be assigned to C2, while those aged 13–24 months tended to belong to C3/C4 (relative to C1), suggesting that different age ranges may correspond to variations in obstruction site, retention duration, and complication evolution pathways. This aligns with epidemiological data indicating that foreign‐body aspiration peaks in the 1‐ to 3‐year age group (approximately 12–36 months) [21, 22].

Regarding clinical clues, a definite history of aspiration and asymmetric breath sounds were more commonly associated with typical airway‐obstruction phenotypes, particularly C3, further supporting the recognized diagnostic value of abnormal or asymmetric breath sounds in pediatric foreign‐body aspiration. Previous studies have also shown that an atypical aspiration history or delayed recognition is associated with a significantly higher incidence of pulmonary complications, especially pneumonia and atelectasis, which may help explain why C1 and C2 were more strongly characterized by inflammatory signals and infection‐related manifestations [23].

Finally, a higher NEUT percentage in C4 than in C3 suggests a more pronounced neutrophil‐driven inflammatory background in phenotypes prone to stenosis. This finding is biologically plausible given the documented progression from chronic or long‐standing foreign‐body retention to granulation tissue proliferation and, ultimately, fixed airway stenosis, further underscoring the importance of early recognition, prompt removal, and timely follow‐up intervention in children at risk of stenosis [24, 25].

### 4.3 Pneumonia–Atelectasis Group Had Higher Healthcare Resource Utilization

A comparative analysis of healthcare resource utilization across the latent classes of pediatric BFB complications showed that children in C2 had longer hospital stays, more days of antibiotic use, and higher treatment costs than those in the other groups. This pattern may be closely related to the clinical characteristics and greater treatment complexity of children with pneumonia–atelectasis. Foreign bodies in the pediatric airway often cause localized airway obstruction, which can lead to recurrent infection, atelectasis, and pneumonia, thereby complicating the clinical course, delaying recovery, and significantly prolonging hospitalization [26]. Because children have relatively narrow airways and immature immune systems, retained foreign bodies are more likely to trigger severe respiratory infections and often require repeated bronchoscopic examinations and foreign body removal procedures. These interventions not only increase treatment costs but may also prolong antibiotic use because of secondary infections [27]. In addition, children generally tolerate medical procedures less well and often require longer postoperative recovery, further increasing healthcare resource consumption. From the perspective of healthcare utilization, the longer length of hospital stay, higher treatment costs, and greater antibiotic use observed in the pneumonia–atelectasis group directly reflect the substantial burden that this complication pattern places on the healthcare system [11].

Upon admission, particular attention should be paid to the child's age, history of foreign body aspiration, presence of asymmetric breath sounds, and levels of CRP and NEUT percentage during both history taking and initial evaluation, as these factors may help predict the potential latent class of complications. As shown in Table 4, the differences in costs among C1–C4 were substantial, with the difference between C1 and C3 exceeding CNY 20,000. These findings suggest that clinicians should establish the diagnosis as early as possible and promptly remove the foreign body through appropriate imaging examinations and bronchoscopic intervention. More refined grouping based on complication clusters may facilitate more precise management at both the patient and hospital levels, thereby reducing the occurrence of complications and avoiding excessive use of medical resources while maintaining quality of care and meeting Diagnosis‐Related Group (DRG) payment requirements [28]. In addition, health education for both parents and healthcare professionals remains essential. Improving awareness of the early recognition and prevention of pediatric BFBs may help reduce the overall disease burden.

### 4.4 Limitations

This study has several limitations. First, it's a single‐center and retrospective study so the results may be influenced by local practice patterns and patient demographics, potentially limiting the generalizability. Second, the sample size was relatively small in groups C1, C2, and C4, which may affect the stability of the estimates. Third, the lack of systematic long‐term follow‐up may lead to an underestimation of late complications, such as the progression of bronchial stenosis or the development of chronic respiratory sequelae, which can manifest months or years after the initial injury [16, 24]. Therefore, further multicenter, prospective studies with larger sample sizes and structured long‐term follow‐up are warranted to validate our findings and enhance their external validity.

## 5 Conclusion

This study employed LCA to identify four potential classes of complications in children with BFBs. Significant differences were observed among the characteristics of these classes, and class membership was associated with age, a confirmed history of foreign body aspiration, asymmetric breath sounds, CRP ≥ 8 mg/L, and neutrophil percentage. Healthcare professionals should pay particular attention to the Pneumonia‐Atelectasis Group, as this phenotype showed higher healthcare resource utilization in terms of hospital stay, treatment costs, and antibiotic used days, and refined and individualized treatment strategies may help improve clinical outcomes and reduce medical costs.

## Author Contributions

**Yong Qian:** data collection, data management, and drafting of the manuscript. **Ling Wang:** study design, research supervision, and manuscript revision. **Chao Niu:** research supervision, manuscript review and revision, and funding support. **Donghai Wang:** research supervision, manuscript review and revision, and funding support. **Yetao Luo:** data management, and statistical analysis.

## Funding

This work was supported by National Outstanding Youth Science Fund Project of National Natural Science Foundation of China (81600022) and the General Project of the Natural Science Foundation of Chongqing (CSTB2024NSCQ‐MSX0281).

## Ethics Statement

This study was approved by the ethics committee of Children's Hospital of Chongqing Medical University (approval number: 2024‐205).

## Conflicts of Interest

Chao Niu is a member of Pediatric Discovery Editorial Board. To minimize bias, he was excluded from all editorial decision‐making related to the acceptance of this article for publication. The other authors declare no conflict of interests.

## Supporting information


Supporting Information S1

## Data Availability

Details of the study design, statistical analysis plan and raw data will be available upon reasonable request to the corresponding author.
