# Supplementary material for: Latent Class Analysis of Pediatric Bronchial Foreign Body Complications and Their Associated Factors
Source: Pediatr Discov. 2026 Sep 23;4(3):e70074. doi: 10.1002/pdi3.70074 (PMC13600466; doi:10.1002/pdi3.70074)
Supplement: Supplementary file 1 [file PDI3-4-e70074-s001.docx]

**Supplementary Table 1 Fit Indices of the Latent Class Model for the Characteristics of Pediatric Bronchial Foreign Body Complications**

| Number of latent classes | AIC | BIC | aBIC | Entropy  index | BLRT  test | LMRT  test | Number of cases (%) in each latent class | Average posterior probability |
| --- | --- | --- | --- | --- | --- | --- | --- | --- |
| 1 | 1657.1 | 1677.9 | 1658.9 | — | — | — | C1=243(100.0%) | C1=1.0 |
| 2 | 1612.1 | 1657.3 | 1616.1 | 0.54 | <0.01 | 0.08 | C1=140(58.8%) C2=98(41.2%) | C1=0.9 C2=0.8 |
| 3 | 1588.3 | 1657.7 | 1594.3 | 0.78 | <0.01 | 0.04 | C1=108(45.4%) C2=33(13.9%) C3=97(40.7%) | C1=1.0 C2=0.8 C3=0.9 |
| 4 | 1581.0 | 1674.8 | 1589.2 | 0.83 | <0.01 | 0.02 | C1=40(16.8%) C2=33(13.9%) C3=106(44.5%) C4=59(24.8%) | C1=0.8 C2=0.9 C3=0.9 C4=1.0 |
| 5 | 1588.1 | 1706.1 | 1598.4 | 0.84 | 0.60 | 0.10 | C1=40(16.8%) C2=32(13.5%) C3=106(44.5%) C4=33(13.9%) C5=27(11.3%) | C1=0.9 C2=1.0 C3=0.8 C4=0.9 C5=1.0 |

Note: AIC, Akaike Information Criterion; BIC, Bayesian Information Criterion; aBIC, sample-size adjusted Bayesian Information Criterion; LMRT, Lo-Mendell-Rubin Likelihood Ratio Test; BLRT, Bootstrapped Likelihood Ratio Test.

**Supplementary Table 2 Independent Variable Coding in the Multivariable Unordered Multinomial Logistic Regression Model**

| Independent Variable | Variable Coding |
| --- | --- |
| Type of foreign body | Dummy variables were set with “Nuts” as the reference: Nut shells = (Z1=1,Z2=0,Z3=0,Z4=0);  Bones = (Z1=0,Z2=1,Z3=0,Z4=0);  Plastic = (Z1=0,Z2=0,Z3=1,Z4=0);  Others = (Z1=0,Z2=0,Z3=0,Z4=1) |
| Age | Dummy variables were set with “≤12 months ” as the reference: 13~24 months = (Z1=1,Z2=0);  >24 months = (Z1=0,Z2=1,) |
| Method of Admission | Dummy variables were set with “Outpatient Clinic ” as the reference: Emergency = (Z1=1,Z2=0);  Transfer from other hospitals = (Z1=0,Z2=1,) |
| History of foreign body aspiration | Denied = 0; Confirmed = 1 |
| Breath sounds | Symmetrical = 0; Asymmetrical = 1 |
| CRP | <8=0；≥8=1 |
| Lymphocyte percentage | Original value |
| Neutrophil percentage | Original value |
